# Synthesis and Initial Characterization of a Reversible, Selective ^18^F-Labeled Radiotracer for Human Butyrylcholinesterase

**DOI:** 10.1007/s11307-021-01584-2

**Published:** 2021-03-03

**Authors:** Christian Gentzsch, Xinyu Chen, Philipp Spatz, Urban Košak, Damijan Knez, Naoko Nose, Stanislav Gobec, Takahiro Higuchi, Michael Decker

**Affiliations:** 1 Pharmaceutical and Medicinal Chemistry, Institute of Pharmacy and Food Chemistry, Julius-Maximilians-University of Würzburg, Am Hubland, 97074 Würzburg, Germany; 2 Department of Nuclear Medicine, University Hospital of Augsburg, Stenglinstraße 2, 86156 Augsburg, Germany; 3 Department of Nuclear Medicine, University Hospital of Würzburg, Oberdürrbacher Straße 6, 97080 Würzburg, Germany; 4 Comprehensive Heart Failure Center, University Hospital of Würzburg, Am Schwarzenberg 15, 97078 Würzburg, Germany; 5 Chair of Pharmaceutical Chemistry, Faculty of Pharmacy, University of Ljubljana, Aškerčeva cesta 7, SI-1000 Ljubljana, Slovenia; 6 Graduate School of Medicine, Dentistry and Pharmaceutical Sciences, Okayama University, 2-5-1 Shikata-cho, Kita-ku, Okayama, Japan

**Keywords:** Alzheimer’s disease, Enzyme inhibitor, Positron emission tomography, Biodistribution, Quaternization

## Abstract

**Purpose:**

A neuropathological hallmark of Alzheimer’s disease (AD) is the presence of amyloid-β (Aβ) plaques in the brain, which are observed in a significant number of cognitively normal, older adults as well. In AD, butyrylcholinesterase (BChE) becomes associated with A_β_ aggregates, making it a promising target for imaging probes to support diagnosis of AD. In this study, we present the synthesis, radiochemistry, *in vitro* and preliminary *ex* and *in vivo* investigations of a selective, reversible BChE inhibitor as PET-tracer for evaluation as an AD diagnostic.

**Procedures:**

Radiolabeling of the inhibitor was achieved by fluorination of a respective tosylated precursor using K[^18^F]. IC_50_ values of the fluorinated compound were obtained in a colorimetric assay using recombinant, human (*h*) BChE. Dissociation constants were determined by measuring *h*BChE activity in the presence of different concentrations of inhibitor.

**Results:**

Radiofluorination of the tosylate precursor gave the desired radiotracer in an average radiochemical yield of 20 ± 3 %. Identity and > 95.5 % radiochemical purity were confirmed by HPLC and TLC autoradiography. The inhibitory potency determined in Ellman’s assay gave an IC_50_ value of 118.3 ± 19.6 nM. Dissociation constants measured in kinetic experiments revealed lower affinity of the inhibitor for binding to the acylated enzyme (*K*_2_ = 68.0 nM) in comparison to the free enzyme (*K*_1_ = 32.9 nM).

**Conclusions:**

The reversibly acting, selective radiotracer is synthetically easily accessible and retains promising activity and binding potential on *h*BChE. Radiosynthesis with ^18^F labeling of tosylates was feasible in a reasonable time frame and good radiochemical yield.

## Introduction

**Fig. 1.**
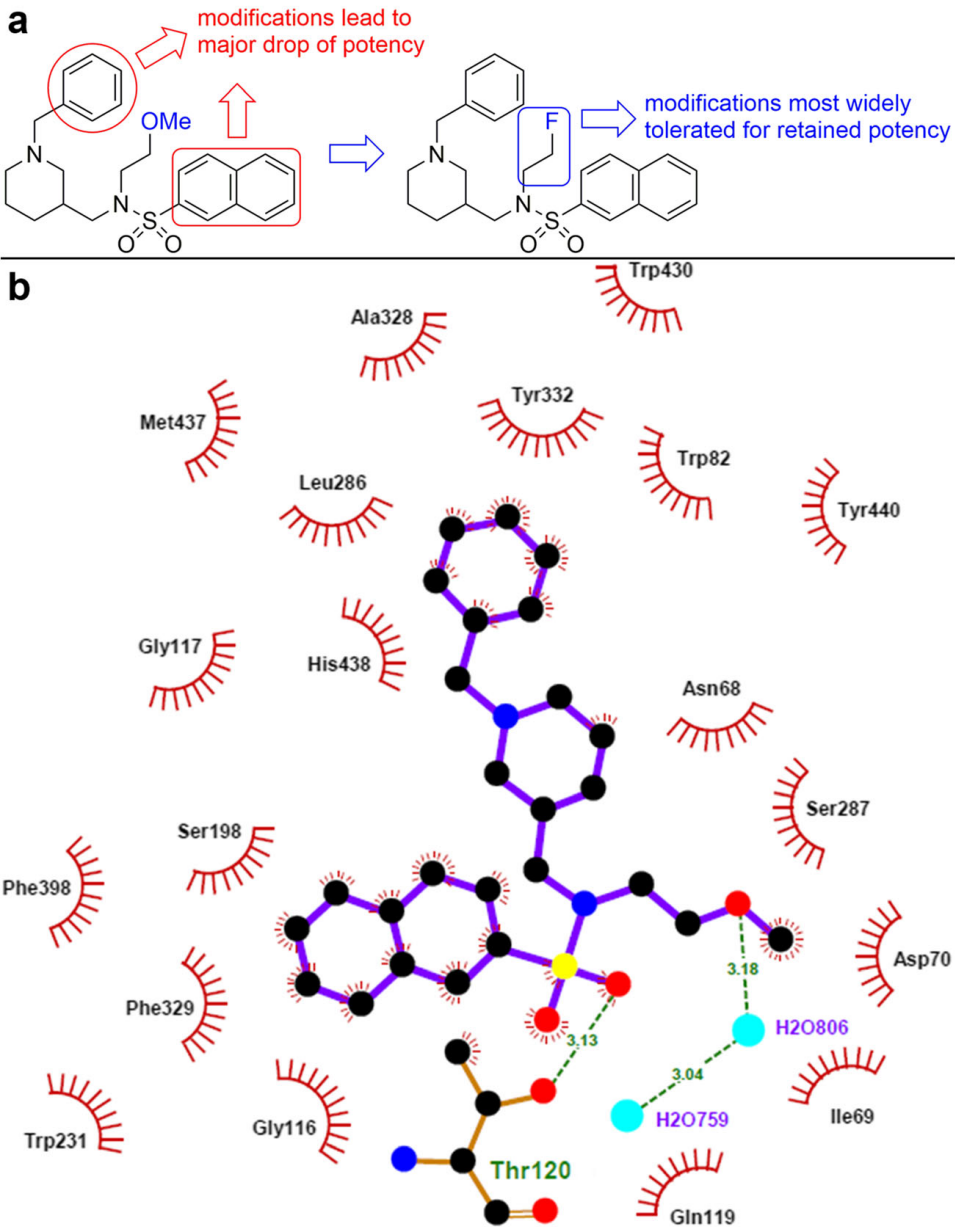
**a** Design of the radiotracer. A potent, selective, and reversibly acting BChE inhibitor served as parent compound [32]. **b** Parent ligand in the BChE binding pocket [32].

Alzheimer’s disease (AD) is an incurable, progressive neurodegenerative disorder and the most frequent cause of dementia with high prevalence and a long asymptomatic phase [1, 2]. The number of dementia-diseased individuals is expected to further increase, due to a growing average age and demographic change, which is hard to overcome for health care systems and society as a whole [3]. The definitive diagnosis of AD should no longer be solely based on typical, clinical symptoms, since they are not conclusive enough and begin many years after initial neuropathological alterations [4–6]. At this point, a successful disease-modifying or even preventive therapy is already impossible. Due to this fact, there is an urgent need for biological targets and biomarkers of an early disease state to help not only for an early, conclusive diagnosis with respective imaging probes but also to shed new light in the sequences and significances of the various neuropathological processes involved [7]. Three of the four FDA-approved anti-AD medications temporarily ameliorate AD symptoms by inhibiting cholinesterases (ChEs), whereby donepezil and galantamine are selective acetylcholinesterase (AChE) inhibitors and rivastigmine additionally binds to the isoenzyme butyrylcholinesterase (BChE). AChE inhibition prevents degradation of the neurotransmitter acetylcholine, levels of which are decreasing through cell death of cholinergic neurons [8–10]. However, advanced progression of AD involves a rapid decrease of AChE levels in the brain of ~ 90 %, which is accompanied in later-stage AD by elevated BChE expression. AChE and BChE exhibit very different localisations, biochemical features, and physiological functions [11]; however, BChE can take over the hydrolytic function of AChE [12–16]. These findings make BChE an attractive target for the development of more potent drugs for combatting AD [8–10]. Established pathological AD hallmarks are the abnormal metabolism of amyloid-β (Aβ), accompanied by hyperphosphorylated tau (τ) proteins, oxidative stress, and alterations concerning microglia cells in nervous tissue [17]. Aβ peptide can impair neurovascular homeostasis by its cerebrovascular effects, causing endothelial dysfunction [18]. Interestingly, Aβ deposits develop in nearly 30 % of aged adults, who do not show pathologically deteriorated cognition or memory deficits [19]. However, in AD patients, there is an increased expression of BChE along with Aβ plaques, especially in the cerebral cortex [20, 21]. In this outer layer of cerebrum, there is usually no considerable amount of BChE in healthy people, suggesting that the enzyme plays a potential key role in generating the detrimental attributes of Aβ. This finding has been supported in a BChE knockout mouse model, where distinctly fewer fibrillar Aβ plaques have been detected [15]. In this context, it is highly promising to apply suitable radiotracers in positron emission tomography (PET) studies, since this imaging technique exhibits excellent sensitivity and can provide an accurate estimation of the radiotracers´ *in vivo* concentration and biodistribution [22]. ChE PET tracers have been developed, with the majority of them targeting peripheral or brain AChE [23]. These compounds can be classified in two main categories, substrate-based and ligand-based tracers. The latter can be reversible or irreversible inhibitors. Substrate-based tracers, which usually have a high blood-brain barrier (BBB) permeability, can have intrinsic drawbacks due to quick hydrolysis and delivery limitations into the tissue [24]. In consequence, they may not reflect the regional enzyme distribution, but rather plasma delivery rate of the tracer [25]. Irreversible inhibitor-derived tracers can have a complex mechanism of enzyme inactivation and it is often complicated to accurately determine *in vivo* kinetics and distribution [24, 26]. Examples of substrate-type tracers specific for BChE include *N*-methylpiperidin-4-yl 4-[^123^I]iodobenzoate ([^123^I]MP4Bz), *N*-[^18^F]fluoroethylpiperidin-4-ylmethyl butyrate, and 1-[^11^C]-methyl-4-piperidinyl *n*-butyrate ([^11^C]MP4B) [16, 27, 28]. [^11^C]MP4B enters the brain, but no enhanced activity can be seen in regions where BChE-associated plaques typically show up in AD, which is likely the same for *N*-[^18^F]fluoroethylpiperidin-4-ylmethyl butyrate. In preliminary *in vivo* studies, the tracer showed high initial uptake in rat cerebral cortex after intravenous injection, but the hydrolysis rate of these ester is presumably still too high [28]. [^123^I]MP4Bz was applied in single-photon emission computed tomography (SPECT) studies, in which it was possible to distinguish cerebral cortical BChE activity in wild-type mice from an AD model [16]. This represents an important proof of concept, which demonstrates that BChE can indeed serve as a promising biomarker in AD diagnostics [15, 21]. This could also be shown for the pseudoirreversible BChE inhibitor phenyl 4-^123^I-iodophenylcarbamate (^123^I-PIP), which accumulated specifically in Aβ plaques with ChE activity in human AD brain tissue [29]. Additionally, this tracer has been investigated very recently as a potential diagnostic and treatment monitoring tool for BChE activity changes in multiple sclerosis showing promising preliminary results [30]. Regarding irreversible and highly selective inhibitors of BChE as radiotracers, carbamate-based inhibitors were investigated that transfer the radiolabeled moiety onto the enzyme, where it is covalently bound. *Ex vivo* autoradiography on mice brain tissue and kinetic investigations proved such covalent transfer [24]. Furthermore, investigations into the influence of the carbamate structure that is transferred to BChE were made by altering spacer lengths and attached heterocyclic moieties. This resulted in sets of inhibitors with short, medium and long duration of action and pronounced neuroprotectivity in an AD mouse model, showing also the therapeutic potential of such inhibitors [31]. Altogether, these results support BChE not only as a promising biomarker for an early diagnosis of AD but also as an attractive target to be addressed by imaging probes in order to shed the light on the complex neuropathology of AD. Herein, we report on synthesis, *in vitro* evaluation, radiolabeling, *ex vivo* autoradiography, and preliminary *in vivo* PET studies of an ^18^F-labeled BChE selective tracer, based on the structure of a potent, selective BChE inhibitor with reversible binding mode (Fig. 1a, b) [32]. Our intention was to utilize the promising properties of the parent compound for a suitable radiotracer, and these properties include the high affinity and selectivity towards the target enzyme, accompanied by pronounced lipophilicity and a relatively low molecular weight, which is favorable for blood-brain barrier (BBB) penetration [33]. Additionally, we chose a reversible inhibitor to overcome the intrinsic problems of substrate-type and irreversible radiotracers mentioned before, possibly enabling a more precise mapping of BChE distribution. We decided to replace the methoxy group on the ethylene-side chain with fluorine, because this moiety points out of the binding pocket as observed in the resolved crystal structure of the enzyme in complex with the parent inhibitor [32]. We assumed that this modification would retain most of inhibitory potency. We chose to incorporate ^18^F as radioisotope to take advantage of a long half-life and high positron yields in combination with a good spatial resolution due to relatively low positron energies in comparison to other radioisotopes commonly used for PET studies (*e.g.*, ^18^F: 0.65 MeV, ^68^Ga: 1.90 MeV) [34, 35].

## Materials and Methods

### Chemistry

**Fig. 2.**
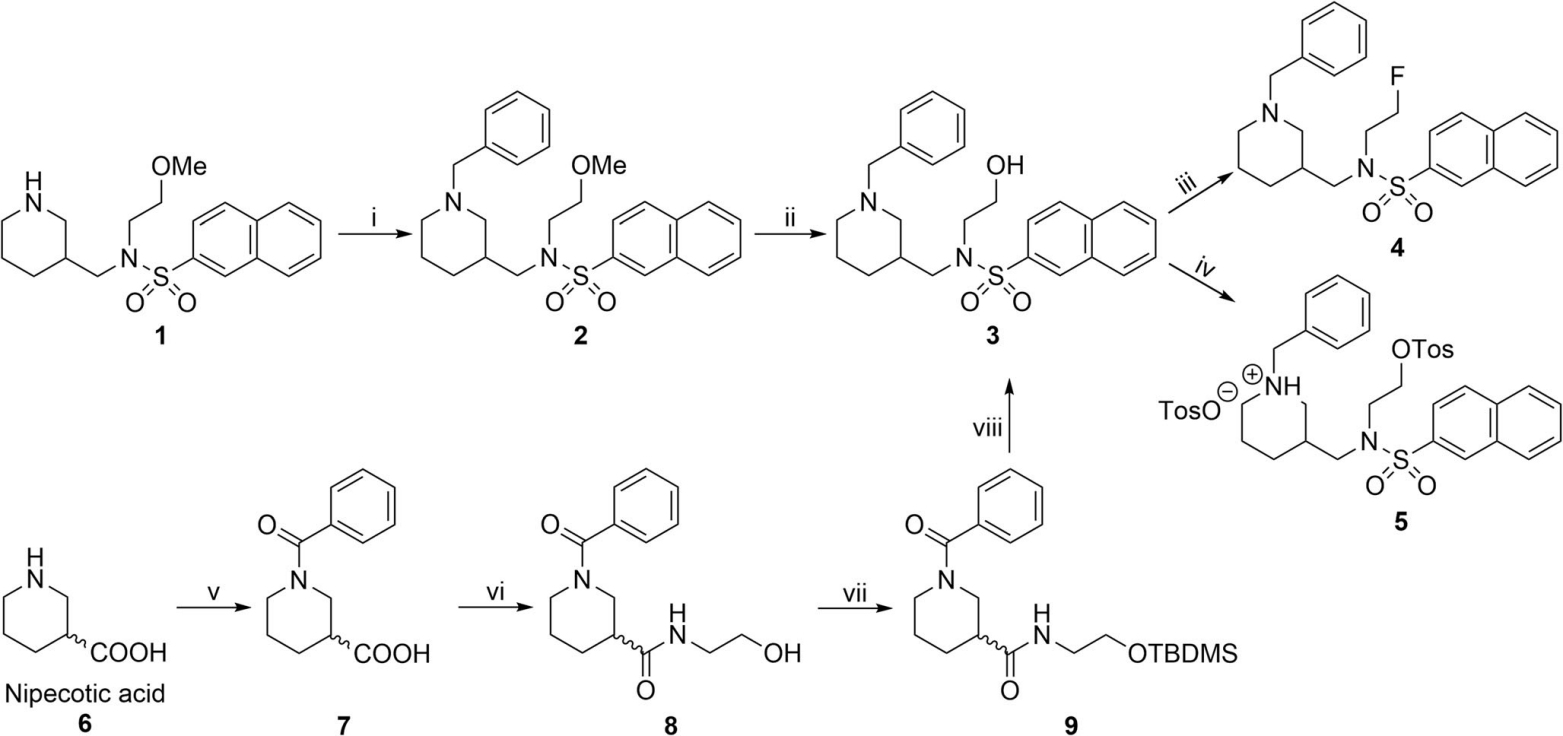
Synthesis of precursor **5** and cold, reversible inhibitor of *h*BChE **4**. Reaction conditions and yields: (i) benzaldehyde, formic acid, 6 h, 180 °C, 44 %; (ii) BF_3_ · Et_2_O, propane-1-thiol, 50 °C, 60 h, 60 %; (iii) DAST, CH_2_Cl_2_, − 10 °C → RT, 24 %; (iv) 1. TosCl, NEt_3_, CH_2_Cl_2_, RT, 18 h, 98 %, 2. *p*-TosOH, MeOH; (v) benzoyl chloride, K_2_CO_3_, THF/H_2_O, 0 °C – RT, 20 h, 88 %; (vi) ethanolamine, HBTU, NEt_3_, DMF, RT, 18 h, 88 %; (vii) TBDMS-Cl, imidazole, DMF, RT, 24 h, 69 %; (viii) 1. LiAlH_4_, THF, reflux, 1 h; 2.naphthalene-2-sulfonyl chloride, DIPEA, CH_2_Cl_2_, 0 °C – RT, 54 % (two steps).

The non-radioactive “cold” inhibitor **4** and precursor **5** for radiolabeling were synthesized as shown in Fig. 2. Briefly, the piperidine ring of building block **1** was benzylated under Leuckart-Wallach conditions [36]. Subsequent demethylation of the methoxy group in compound **2** applying BF_3_ · Et_2_O in propane-1-thiol [37] gave the central building block, the alcohol **3**, which can also be obtained from commercially available nipecotic acid (Fig. 2) [38]. Cold compound **4** was obtained by fluorination of **3** with diethylaminosulfur trifluoride (DAST) [39]. For radiolabeling with K[^18^F], a tosylate-leaving group was chosen, which was introduced by reacting **3** with *p*-toluenesulfonyl chloride (TosCl). We chose this synthetic strategy to utilize the advantage of having one central building block for generating both the precursor **5** for radiolabeling and the cold reference compound **4**.

### *In Vitro* Studies

The inhibitory potency of compound **4** (Fig. 2) on *h*BChE was determined in the colorimetric Ellman’s assay [40]. The incubation time of the compound stock solution (100 % in dimethylsulfoxide, DMSO) with Ellman’s reagent (5,5′-dithiobis-(2-nitrobenzoic acid), DTNB) and recombinant *h*BChE was 5 min. The final concentrations were 370 μM of DTNB and 1 nM of the enzyme in a 0.1-M phosphate buffer at pH = 8.0. Reactions were started by addition of butyrylthiocholine iodide (BTCI) in a concentration of 500 μM with a final DMSO content of 1 %. IC_50_ values were determined by plotting residual enzyme activities against seven respective inhibitor concentrations. Tacrine served as a positive control (*cf.* ESM for further experimental details).

Kinetic studies to measure dissociation constants *K*_1_ and *K*_2_ of inhibitor **4** for binding to the free and acylated enzyme were performed by measuring progress curves of product formation with ~ 50 μM BTCI as substrate. BTCI hydrolysis by *h*BChE was measured in the absence and presence of inhibitor **4** at three different concentrations (namely 40 nM, 80 nM, and 160 nM, respectively). The experiments were carried out at 25 °C in 25-mM phosphate buffer (pH = 7.0) according to the method of Ellman [40]. The concentration of purified *h*BChE, which was always the final addition to the assay probes, was approx. 1 nM. The hydrolysis of 46 μM BTCI was followed until completion in the presence of 1 mM DTNB (*cf.* ESM for further experimental details).

### Radiochemistry

Radiofluorination of tosylate precursor **5** (Fig. 2) was performed in an established procedure. ^18^F-Fluoride was separated from ^18^O-water by an anion-exchange cartridge, which was eluted with 300 μl of 66-mM K_2_CO_3_ aqueous solution into a v-vial containing 15 mg of Kryptofix_222_ in 500-μl acetonitrile. The mixture was dried azeotropically at 120 °C, which was repeated twice using dry acetonitrile (500 μl each time). Then, 1 mg of the precursor **5** (Fig. 2) in 400 μl of dry acetonitrile was added to the mixture and reacted for 10 min at 110 °C. After cooling to room temperature, the reaction mixture was neutralized by adding 5 % acetic acid (300 μl). The labeled compound was purified by semi-preparative, reversed phase HPLC (*cf.* ESM for further experimental details). Tracer identity and sufficient radiochemical purity were confirmed by radio-thin layer chromatography (TLC). The radiotracer was diluted with saline to the required concentration for further investigation.

### Preliminary *Ex Vivo* Tissue Binding Assay and *In Vivo* PET Imaging

Animal protocols were approved by the local Animal Care and Use Committee and conducted according to the Guide for the Care and Use of Laboratory Animals. One C57BL/6N mouse from Charles River was used for *ex vivo* tissue binding studies. Horizontal slices with 20-μm thickness were made and separated into two series for either control or blocking group. The frozen mice brain slices were incubated in a buffer at pH = 8.0 (150-mM NaCl, 5-mM EDTA, 50-mM Na_2_HPO_4_) containing ^18^F-labeled compound **4** (*A* = 1.44 MBq) with or without ethopropazine hydrochloride (60 μM) as blocking agent. After incubating for 30 min at 25 °C, the slices were rinsed five times in PBS buffer (1 min each time). After drying at room temperature, the slices were exposed to a phosphor imaging plate (Fuji SR-type image plate, Fujifilm Corporation, Tokyo, Japan). The images were obtained using a digital autoradiographic system (Typhoon FLA 7000). For *in vivo* PET imaging, a healthy male Wistar rat, which was anesthetized and maintained with isoflurane, was scanned using a micro-PET system (FOCUS, Siemens). Directly after the injection of tracer **4** (6.3 MBq), a 60-min dynamic imaging protocol was started. The obtained PET images were analyzed with the public domain tool AMIDE imaging software (A Medical Imaging Data Examiner, version 1.01) and used to generate time-activity curves of regions of interest.

## Results

### Chemistry

**Fig. 3.**
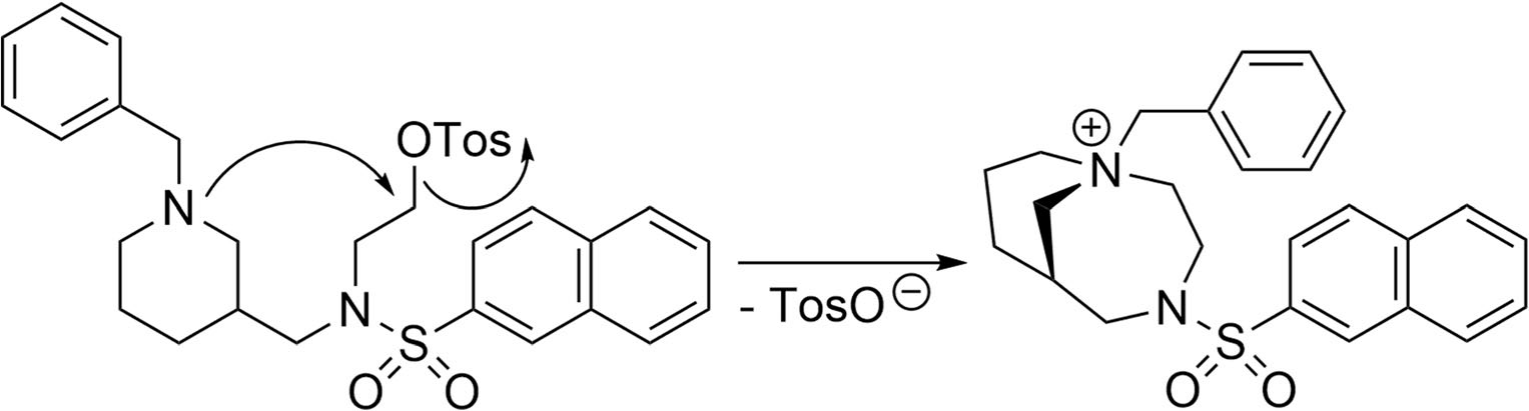
Quaternization of precursor **5** when present as free base.

The fluorinated, reversible *h*BChE inhibitor **4** (Fig. 2) was synthesized in three steps with satisfying yields [36, 37, 39]. We applied the DAST fluorination method for alcohol **3** due to the short reaction times, mild conditions, and uncomplicated workup, despite lower yields (24 %). This compound was only required in minor amounts as reference during radiolabeling and for *in vitro* assays. The respective precursor for ^18^F-labeling was synthesized using the same building block, alcohol **3**, in almost quantitative yields. However, precursor **5** exhibited an instability problem, when present as free base. It was resolved by turning **5** into its tosylate salt. During purity control by liquid chromatography/mass spectrometry (LCMS), we found a slow side reaction due to intramolecular ring closure by nucleophilic attack of the piperidine nitrogen (Fig. 3). This can be explained with the excellent leaving group quality of tosylate, which is on the one hand required for a facile radiolabeling under preferably mild conditions, but makes the compound sensitive for this quaternization side reaction on the other hand. Since building block **1** is not commercially available, we synthesized alcohol **3** additionally out of low cost and easy to handle nipecotic acid **6** (Fig. 2) [36, 37]. In the first step, the piperidine nitrogen was benzoylated in very good yields applying benzoyl chloride. Next, the carboxylic acid of compound **7** was activated with 3-[bis(dimethylamino)methyliumyl]-3*H*-benzotriazol-1-oxide hexafluorophosphate (HBTU) and coupled to 2-aminoethanol in very good yields. Subsequently, compound **8** was protected at its hydroxyl group with tert-butyldimethylsilyl chloride (TBDMS-Cl) in good yields. Finally, both amide groups were reduced with lithium aluminum hydride and the crude product was used directly in a one-pot-two-steps manner to be coupled naphthalene-2-sulfonyl chloride at the reduced secondary amine function. It was found that the TBDMS group is cleaved under the applied conditions and alcohol **3** (Fig. 2) was obtained in satisfying yields.

### *In Vitro* Studies

Subsequently, compound **4** was tested in an Ellman’s assay for its inhibitory potency against *h*BChE [40]. We determined a low submicromolar value (IC_50_ = 118.3 ± 19.6 nM), meaning a drop in inhibitory potency compared to the parent methoxy derivative (Fig. 1a, b; IC_50_ = 4.9 ± 0.3 nM) [32]. This can be explained by means of the BChE crystal structure in complex with the inhibitor. Although the methoxy-ethylene moiety as a whole rather points out of the binding pocket, the missing methoxy-oxygen has been described to act as an additional H-bond acceptor with a structural water and Asn68 (Fig. 1b). This is still a good compromise, since the structure-activity relationships revealed that other positions to introduce fluorine in the molecule would presumably lead to a more drastic decline of activity (*cf.* Table 1). Still, this class of compounds exhibits high selectivity over *h*AChE, which had been described also for several derivatives thereof [32].

**Table 1:**
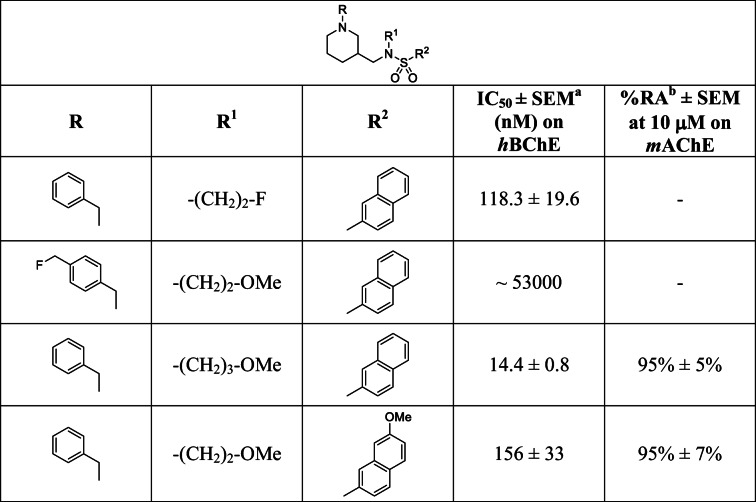
Structures and inhibitory potencies of cold, reversible *h*BChE inhibitor **4** (Fig. 2) in contrast to respective derivatives with substituted benzyl group, naphthalene group, and altered alkyl chain length on sulfonamidic nitrogen [32]. ^**a**^SEM standard error of means, ^**b**^RA residual activity

**Fig. 4.**
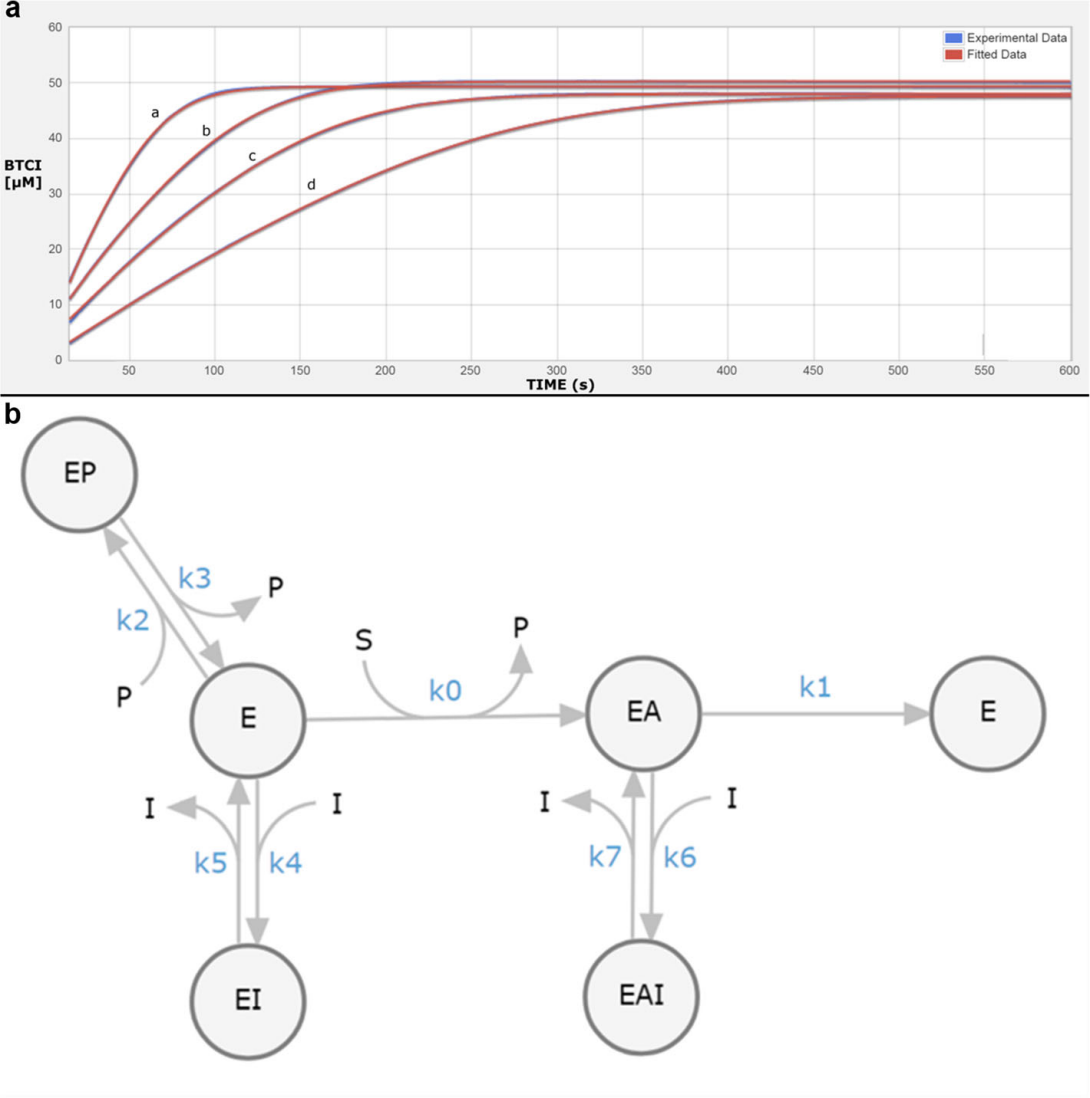
**a** Time courses of product formation in the reactions between BTCI (~ 46 μM) and purified *h*BChE (1 nM) in the absence (curve a) and presence of compound **4** (b, 40 nM; c, 80 nM; d, 160 nM). **b** Scheme for the inhibition of BTCI hydrolysis by *h*BChE in the presence of DTNB by compound **4**. E, free enzyme; EA, acylated intermediate; S, substrate (BTCI); P, all of the stoichiometrically released products (thiocholine-TNB, TNB^−^); and I, compound **4** (inhibitor). The symbols for the constants are *k*_cat_ (*k*_1_), catalytic constant for BTCI turnover; *K*_m_ (*k*_0_), Michaelis constant; *K*_p_ = *k*_3_/*k*_2_, inhibition constant for binding of the product thiocholine-TNB; *K*_1_ = *k*_5_/*k*_4_ and *K*_2_ = *k*_7_/*k*_6_, dissociation constants for binding of the compound to the free and acylated enzyme, respectively.

To gain in-depth insight into the binding potential of inhibitor **4**, the effects of different concentrations of the compound on *h*BChE activity were studied by measuring progressive curves of product formation at approximately 50 μM of BTCI (Fig. 4a). The analysis of these progressive curves revealed good agreement between the experimental curves (Fig. 4a, in blue) and a theoretical model (Fig. 4a, in red) that defined a mixed reaction mechanism with binding of the inhibitor to both the free and the acylated enzyme (Fig. 4b). The dissociation constants revealed that the binding affinity of compound **4** to the acylated enzyme (*K*_2_ = 68.0 nM) is lower than to the free enzyme (*K*_1_ = 32.9 nM).

### Radiochemistry

**Fig. 5.**
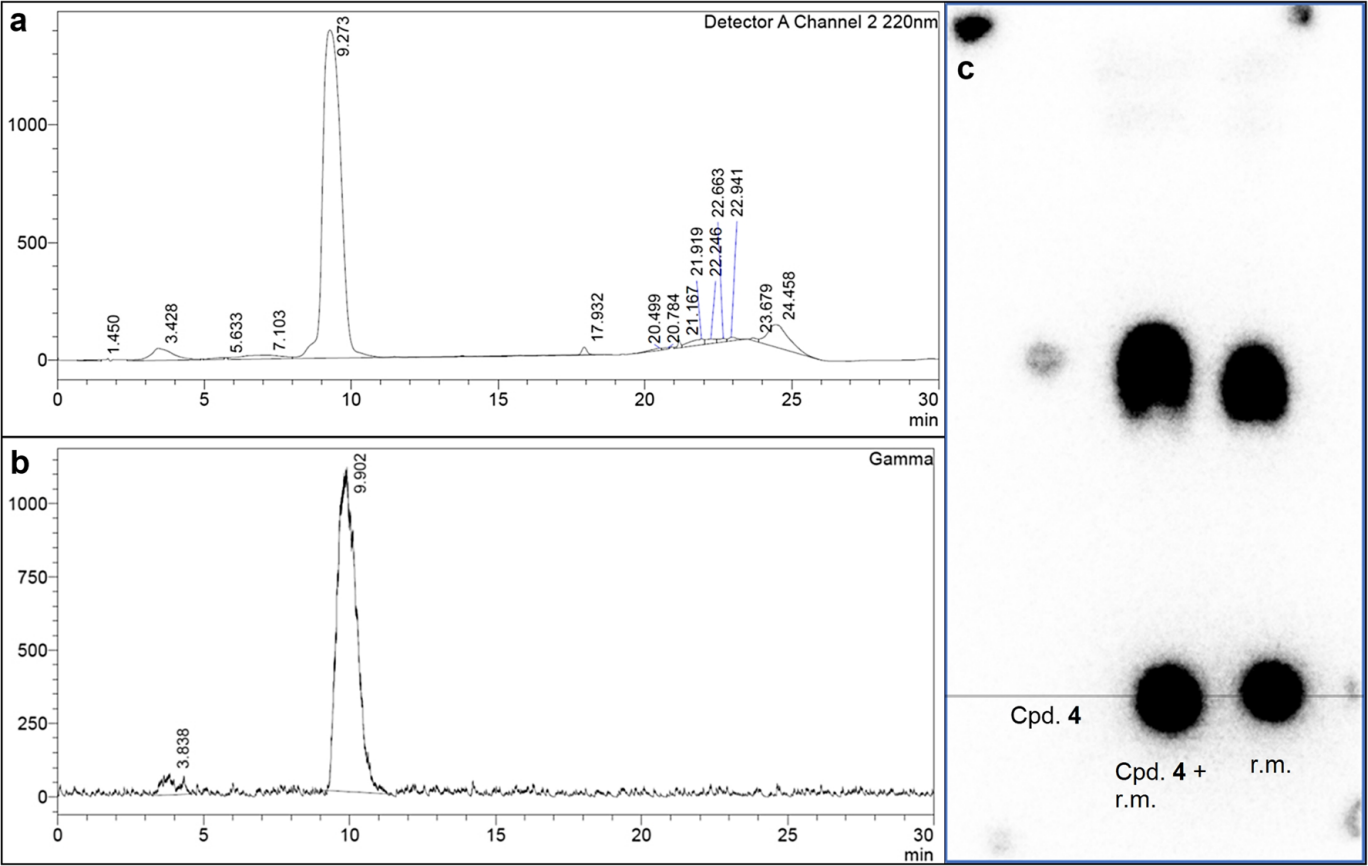
HPLC – and radio-TLC analysis after first ^18^F radiolabeling of precursor **5**. **a** HPLC chromatogram of reference compound **4**. The UV absorption at 220 nm was detected. **b** HPLC chromatogram of the purified radiotracer. γ-ray emission produced by the β^+^-(positron-) decay of ^18^F and subsequent annihilation with electrons was detected. The retention time (Rt) is in good agreement with the reference (cold compound: Rt = 9.273 min; radiotracer: Rt = 9.902 min). **c** Radio-TLC analysis of labeling progress. The *R*_f_ values of cold compound (cpd. **4**) and the respective spot of the tracer in the reaction mixture (r. m.) are in good agreement. TLC autoradiography indicated a process of fluorination by more than 33 %.

Next, precursor **5** (Fig. 2) was subjected to ^18^F-radiolabeling. We obtained [^18^F]-labeled tracer **4** in approximately 120 min with an average radiochemical yield of 20 ± 3 % (decay-corrected, *n* = 2) without reaction condition optimization. The identity of the tracer and its radiochemical purity (≥ 95.3 %) were confirmed by TLC autoradiography. In a preceding ^18^F radiolabeling approach of precursor **5** applying established conditions as described before (*cf.* “Materials and Methods”), we were able to verify tracer identity by its *R*_f_ value on radio-TLC. TLC autoradiography indicated a progress of fluorination by more than 33 % (Fig. 5c). Furthermore, the retention time of the radiotracer (γ detection, Fig. 5b) corresponded to that of the cold compound on HPLC (UV detection, Fig. 5a).

### Preliminary *Ex Vivo* Tissue Binding and *In Vivo* PET Imaging

**Fig. 6.**
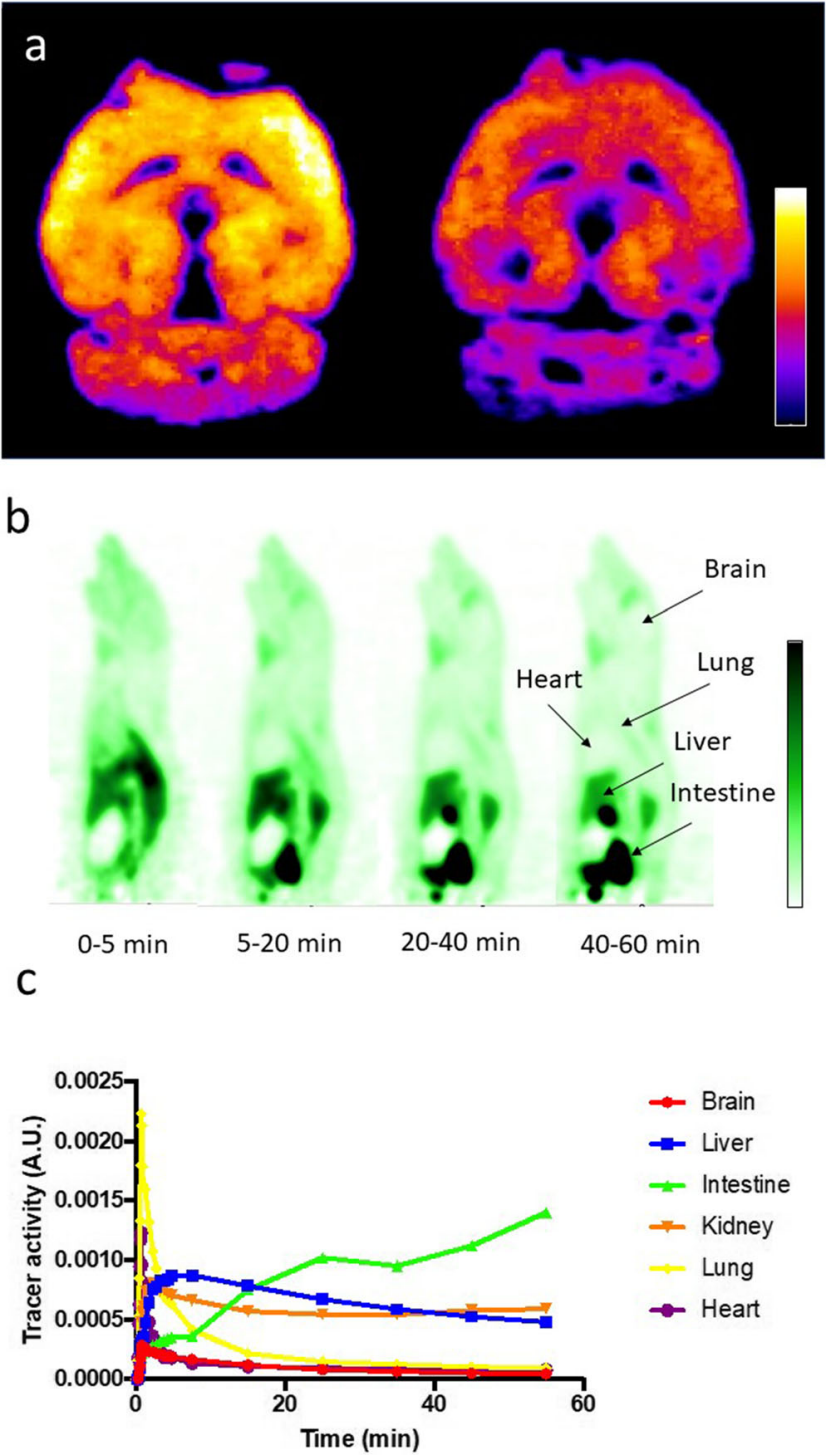
**a** Autoradiographic image of tissue binding assay with healthy mice brain slices incubated with [^18^F]-labeled BChE – tracer **4** without (left) and with (right) ethopropazine. Purple areas represent low binding; bright yellow areas represent high binding. **b** Dynamic PET images of sagittal sections after administration of radiotracer **4** in a healthy rat. Dark green areas represent high tracer accumulation; light green areas represent low accumulation. **c** Time-activity curves in each organ derived from the PET imaging.

In the *ex vivo* autoradiography study, we found accumulation of high radioactivity in most of the brain area, especially high intensity at the cortex, where BChE activity is elevated even more during AD progression [41]. Ethopropazine hydrochloride, a selective and reversible inhibitor of BChE [42], successfully reduced tracer uptake, suggesting specific binding of the tracer to BChE in the brain tissue (Fig. 6a). Unfortunately, dynamic PET images in a healthy rat indicated low tracer retention in brain (Fig. 6b, c).

## Discussion

In our synthetic approach towards cold, reversible *h*BChE inhibitor **4**, and the respective precursor **5** for ^18^F labeling (Fig. 2), we were able to perform several optimizations of reaction conditions. Yields of the Leuckart-Wallach benzylation in the first step were significantly increased, when benzaldehyde was freshly distilled before the reaction to remove benzoic acid, which is formed due to slow oxidation of benzaldehyde in the presence of air [36, 43]. In the next step, we achieved demethylation of a methoxy group with boron trifluoride etherate in propane-1-thiol. However, at first, this step required long reaction times (6–7 days), even though it was described that increased amounts of BF_3_ ∙ Et_2_O can accelerate conversion [37]. We found that slightly elevated temperatures (up to 50 °C) significantly decreased reaction times (60 h). Fluorination of the alcohol **3** with DAST gave the desired product **4**; however, attempts to increase the yields failed. This is likely due to the side reactions that can appear when DAST is applied, namely elimination or carbonium-ion type rearrangements [39]. Tosylation of alcohol **3** proceeded almost quantitatively under standard conditions [44]; however, the product has to be turned into its tosylate salt to prevent slow, but continuous quaternization (Fig. 3). In our additional synthetic approach towards central building block **3** (Fig. 2), we found that both amide groups of TBDMS-protected compound **9** can be reduced by lithium aluminum hydride and the crude product can directly be coupled to naphthalene-2-sulfonyl chloride to obtain deprotected alcohol **3**. This abbreviates the synthetic procedure by one additional TBDMS-deprotection step.

Next, we measured the inhibitory potency of our cold, reversible *h*BChE inhibitor in a colorimetric Ellman’s assay and determined an IC_50_ value of 118.3 ± 19.6 nM, meaning a significant drop of inhibitory activity compared to the parent compound (Fig. 1a, IC_50_ = 4.9 ± 0.3 nM). However, the structure-activity relationships for this class of compounds revealed that altering the *N*-alkyl moieties of the sulfonamide nitrogen led to the lowest changes in inhibitory potency, while substituted naphthalene or benzyl groups significantly decreased inhibitory potency (Table 1 and Fig. 1b) [32]. As an example, we synthesized a respective derivative with a fluoromethyl group in *para* position of the benzyl ring, which turned out to be almost inactive as *h*BChE inhibitor (Table 1). Even though replacement of the methoxy group with a fluorine atom reduced inhibitory potency more than expected considering the previously established structure-activity relationships, the submicromolar IC_50_ value in combination with the high selectivity ratio over AChE for this class of compounds still represent promising attributes for a suitable radiotracer [33]. Additionally, kinetic experiments revealed a good binding potential of compound **4** (Fig. 2) to *h*BChE. The dissociation constant *K*_1_, representing the binding affinity to the free enzyme, was generally lower than *K*_2_, the respective constant for binding to the acylated enzyme (*K*_1_ = 32.9 nM, K_2_ = 68.0 nM, *cf.* Fig. 4).

Subsequently, we performed ^18^F-radiolabeling of the tosylate precursor **5** by nucleophilic substitution. Applying an established procedure with some variations on the technical details led to a reasonable radiochemical yield. Importantly, the whole process including labeling, tracer identification, and purification was feasible in approximately 120 min, considering the half-life of ^18^F (1.8288 h) as a limiting factor for time-consuming preparations of radiotracers [34]. In a precedent radiolabeling approach, we could already determine a facile progress of fluorination by TLC autoradiography. The tracer identity could be confirmed both by radio-TLC and HPLC retention times (Fig. 5). Since harsh radiolabeling conditions can lead to decomposition of unstable functional groups and unexpected side reactions of the respective precursor, our promising radiolabeling results motivated us to perform preliminary *ex vivo* and *in vivo* investigations.

The *ex vivo* autoradiography study demonstrated good binding of our tracer to BChE rich areas in mice brain tissues. We observed high intensities in cortex, which is in good agreement with the known BChE distribution in mice brain [45–47]. After preincubating the tissue with ethopropazine hydrochloride, a selective inhibitor of BChE [42], we found a significant decrease of binding (Fig. 6a). This finding met our expectations and provides further evidence of the pronounced selectivity over AChE for this compound. Nevertheless, a significant amount of tracer remains bound to the tissue despite blocking. Possible reasons are non-specific binding due to the lipophilicity of the tracer and/or potential off-target effects.

However, in our first approach to utilize the tracer for *in vivo* PET studies, we found only low brain uptake after administering the compound *via* tail vein to a male rat (Fig. 6b, c). This can reflect the compounds limited  BBB permeability, since a moderate brain-to-plasma ratio (0.44 *vs.* Donepezil = 6.3) had already been described for the parent, methoxy compound (Fig. 1a) after *in vivo* blood plasma-brain distribution studies [32]. On the other hand, this compound had been investigated in permeability measurements using Caco-2 cells, where it exhibited neither low passive permeability nor active efflux by membrane transport proteins like P-glycoprotein or breast cancer resistance protein [32].

## Conclusion

In our study, we present design and evaluation of a novel ^18^F-PET radiotracer selectively targeting BChE with reversible mode of binding. Precursor and the respective cold compound are now synthetically easily accessible. Inhibitory potency of the cold compound was decreased compared to the parent compound. Therefore, future studies could additionally focus on ^11^C labeling at the methoxy group to completely retain inhibitory potency of the radiotracer. Radiolabeling was achieved in a reasonable time frame and a good radiochemical yield applying a standard procedure. Preliminary *ex vivo* autoradiography on mice brain slices preincubated with the tracer revealed its good binding to brain tissue and blocking studies with ethopropazine hydrochloride demonstrated its selectivity towards BChE. However, future studies should focus on determining possible reasons for the significant compound retention despite blocking with respect to potential off-target effects and non-specific binding. Our preliminary *in vivo* PET study showed only limited brain uptake of the tracer after tail vein injection. After the initial blood pool circulation, the tracer was accumulated in liver and kidneys and excreted fast into intestine and urine. The tracer’s ability to pass the BBB might be restricted. On the other hand, the parent compound, which served as design schedule for us, demonstrated neither low passive permeability nor active efflux. Due to these facts, the tracer will be applied in future studies towards its precise biodistribution to clarify the reason of its limited brain uptake.

## References

[1] Masters CL, Bateman R, Blennow K, Rowe CC, Sperling RA, Cummings JL (2015). Alzheimer’s disease. Nat Rev Dis Primers.

[2] Coyle J, Price D, DeLong M (1983). Alzheimer’s disease: a disorder of cortical cholinergic innervation. Science.

[3] Nichols E, Szoeke CEI, Vollset SE, Abbasi N, Abd-Allah F, Abdela J, Aichour MTE, Akinyemi RO, Alahdab F, Asgedom SW, Awasthi A, Barker-Collo SL, Baune BT, Béjot Y, Belachew AB, Bennett DA, Biadgo B, Bijani A, Bin Sayeed MS, Brayne C, Carpenter DO, Carvalho F, Catalá-López F, Cerin E, Choi JYJ, Dang AK, Degefa MG, Djalalinia S, Dubey M, Duken EE, Edvardsson D, Endres M, Eskandarieh S, Faro A, Farzadfar F, Fereshtehnejad SM, Fernandes E, Filip I, Fischer F, Gebre AK, Geremew D, Ghasemi-Kasman M, Gnedovskaya EV, Gupta R, Hachinski V, Hagos TB, Hamidi S, Hankey GJ, Haro JM, Hay SI, Irvani SSN, Jha RP, Jonas JB, Kalani R, Karch A, Kasaeian A, Khader YS, Khalil IA, Khan EA, Khanna T, Khoja TAM, Khubchandani J, Kisa A, Kissimova-Skarbek K, Kivimäki M, Koyanagi A, Krohn KJ, Logroscino G, Lorkowski S, Majdan M, Malekzadeh R, März W, Massano J, Mengistu G, Meretoja A, Mohammadi M, Mohammadi-Khanaposhtani M, Mokdad AH, Mondello S, Moradi G, Nagel G, Naghavi M, Naik G, Nguyen LH, Nguyen TH, Nirayo YL, Nixon MR, Ofori-Asenso R, Ogbo FA, Olagunju AT, Owolabi MO, Panda-Jonas S, Passos VMA, Pereira DM, Pinilla-Monsalve GD, Piradov MA, Pond CD, Poustchi H, Qorbani M, Radfar A, Reiner RC, Robinson SR, Roshandel G, Rostami A, Russ TC, Sachdev PS, Safari H, Safiri S, Sahathevan R, Salimi Y, Satpathy M, Sawhney M, Saylan M, Sepanlou SG, Shafieesabet A, Shaikh MA, Sahraian MA, Shigematsu M, Shiri R, Shiue I, Silva JP, Smith M, Sobhani S, Stein DJ, Tabarés-Seisdedos R, Tovani-Palone MR, Tran BX, Tran TT, Tsegay AT, Ullah I, Venketasubramanian N, Vlassov V, Wang YP, Weiss J, Westerman R, Wijeratne T, Wyper GMA, Yano Y, Yimer EM, Yonemoto N, Yousefifard M, Zaidi Z, Zare Z, Vos T, Feigin VL, Murray CJL (2019). Global, regional, and national burden of Alzheimer’s disease and other dementias, 1990-2016: a systematic analysis for the Global Burden of Disease Study 2016. Lancet Neurol.

[4] Villemagne VL, Burnham S, Bourgeat P, Brown B, Ellis KA, Salvado O, Szoeke C, Macaulay SL, Martins R, Maruff P, Ames D, Rowe CC, Masters CL, Australian Imaging Biomarkers and Lifestyle (AIBL) Research Group (2013). Amyloid β deposition, neurodegeneration, and cognitive decline in sporadic Alzheimer’s disease: a prospective cohort study. Lancet Neurol.

[5] Jack CR, Lowe VJ, Weigand SD (2009). Serial PIB and MRI in normal, mild cognitive impairment and Alzheimer’s disease: implications for sequence of pathological events in Alzheimer’s disease. Brain.

[6] Braak H, Thal DR, Ghebremedhin E, Del Tredici K (2011). Stages of the pathologic process in Alzheimer disease: age categories from 1 to 100 years. J Neuropathol Exp Neurol.

[7] Jack CR, Bennett DA, Blennow K (2018). NIA-AA research framework: toward a biological definition of Alzheimer’s disease. Alzheimers Dement.

[8] Rountree SD, Chan W, Pavlik VN, Darby EJ, Siddiqui S, Doody RS (2009). Persistent treatment with cholinesterase inhibitors and/or memantine slows clinical progression of Alzheimer disease. Alzheimers Res Ther.

[9] Raina P, Santaguida P, Ismaila A, Patterson C, Cowan D, Levine M, Booker L, Oremus M (2008). Effectiveness of cholinesterase inhibitors and memantine for treating dementia: evidence review for a clinical practice guideline. Ann Intern Med.

[10] Nordberg A, Ballard C, Bullock R, Darreh-Shori T, Somogyi M (2013) A review of butyrylcholinesterase as a therapeutic target in the treatment of Alzheimer’s disease. Prim Care Companion CNS Disord 15. 10.4088/PCC.12r01412 10.4088/PCC.12r01412 PMC3733526 23930233

[11] Lockridge O, Duysen E, Masson P (2011) Butyrylcholinesterase: overview, structure, and function. pp 25–41

[12] Arendt T, Brückner MK, Lange M, Bigl V (1992). Changes in acetylcholinesterase and butyrylcholinesterase in Alzheimer’s disease resemble embryonic development--a study of molecular forms. Neurochem Int.

[13] Giacobini E (2003). Cholinergic function and Alzheimer’s disease. Int J Geriatr Psychiatry.

[14] Greig NH, Utsuki T, Yu Q (2001). A new therapeutic target in Alzheimer’s disease treatment: attention to butyrylcholinesterase. Curr Med Res Opin.

[15] Darvesh S (2016). Butyrylcholinesterase as a diagnostic and therapeutic target for Alzheimer’s disease. Curr Alzheimer Res.

[16] DeBay DR, Reid GA, Pottie IR (2017). Targeting butyrylcholinesterase for preclinical single photon emission computed tomography (SPECT) imaging of Alzheimer’s disease. Alzheimers Dement (N Y).

[17] Wang J, Gu BJ, Masters CL, Wang YJ (2017). A systemic view of Alzheimer disease - insights from amyloid-β metabolism beyond the brain. Nat Rev Neurol.

[18] Koizumi K, Wang G, Park L (2016). Endothelial dysfunction and amyloid-β-induced neurovascular alterations. Cell Mol Neurobiol.

[19] Katzman R, Terry R, DeTeresa R, Brown T, Davies P, Fuld P, Renbing X, Peck A (1988). Clinical, pathological, and neurochemical changes in dementia: a subgroup with preserved mental status and numerous neocortical plaques. Ann Neurol.

[20] Reid GA, Darvesh S (2015). Butyrylcholinesterase-knockout reduces brain deposition of fibrillar β-amyloid in an Alzheimer mouse model. Neuroscience.

[21] Macdonald IR, Maxwell SP, Reid GA, Cash MK, DeBay DR, Darvesh S (2017). Quantification of butyrylcholinesterase activity as a sensitive and specific biomarker of Alzheimer’s disease. J Alzheimers Dis.

[22] Vaquero JJ, Kinahan P (2015). Positron emission tomography: current challenges and opportunities for technological advances in clinical and preclinical imaging systems. Annu Rev Biomed.

[23] Holland JP, Liang SH, Rotstein BH, Collier TL, Stephenson NA, Greguric I, Vasdev N (2014). Alternative approaches for PET radiotracer development in Alzheimer’s disease: imaging beyond plaque. J Labelled Compd Rad.

[24] Sawatzky E, Al-Momani E, Kobayashi R (2016). A novel way to radiolabel human butyrylcholinesterase for positron emission tomography through irreversible transfer of the radiolabeled moiety. ChemMedChem.

[25] Logan J, Fowler JS, Ding Y-S, Franceschi D, Wang GJ, Volkow ND, Felder C, Alexoff D (2002). Strategy for the formation of parametric images under conditions of low injected radioactivity applied to PET studies with the irreversible monoamine oxidase a tracers [11C]clorgyline and deuterium-substituted [11C]clorgyline. J Cereb Blood Flow Metab.

[26] Fowler JS, Logan J, Volkow ND, Wang GJ, MacGregor R, Ding YS (2002). Monoamine oxidase: radiotracer development and human studies. Methods.

[27] Roivainen A, Rinne J, Virta J, Järvenpää T, Salomäki S, Yu M, Någren K (2004). Biodistribution and blood metabolism of 1-11C-methyl-4-piperidinyl n-butyrate in humans: an imaging agent for in vivo assessment of butyrylcholinesterase activity with PET. J Nucl Med.

[28] Kikuchi T, Zhang M-R, Ikota N, Fukushi K, Okamura T, Suzuki K, Arano Y, Irie T (2004). N-[18F]fluoroethylpiperidin-4-ylmethyl butyrate: a novel radiotracer for quantifying brain butyrylcholinesterase activity by positron emission tomography. Bioorg Med Chem Lett.

[29] Macdonald IR, Reid GA, Pottie IR, Martin E, Darvesh S (2016). Synthesis and preliminary evaluation of phenyl 4-123I-iodophenylcarbamate for visualization of cholinesterases associated with Alzheimer disease pathology. J Nucl Med.

[30] Thorne MWD, Cash MK, Reid GA, Burley DE, Luke D, Pottie IR, Darvesh S (2020). Imaging butyrylcholinesterase in multiple sclerosis. Mol Imaging Biol.

[31] Hoffmann M, Stiller C, Endres E, Scheiner M, Gunesch S, Sotriffer C, Maurice T, Decker M (2019). Highly selective butyrylcholinesterase inhibitors with tunable duration of action by chemical modification of transferable carbamate units exhibit pronounced neuroprotective effect in an Alzheimer’s disease mouse model. J Med Chem.

[32] Košak U, Brus B, Knez D, Šink R, Žakelj S, Trontelj J, Pišlar A, Šlenc J, Gobec M, Živin M, Tratnjek L, Perše M, Sałat K, Podkowa A, Filipek B, Nachon F, Brazzolotto X, Więckowska A, Malawska B, Stojan J, Raščan IM, Kos J, Coquelle N, Colletier JP, Gobec S (2016). Development of an in-vivo active reversible butyrylcholinesterase inhibitor. Sci Rep.

[33] McCluskey SP, Plisson C, Rabiner EA, Howes O (2020). Advances in CNS PET: the state-of-the-art for new imaging targets for pathophysiology and drug development. Eur J Nucl Med Mol Imaging.

[34] Sanchez-Crespo A (2013). Comparison of Gallium-68 and Fluorine-18 imaging characteristics in positron emission tomography. Appl Radiat Isot.

[35] Kesch C, Kratochwil C, Mier W, Kopka K, Giesel FL (2017). (68)Ga or (18)F for prostate cancer imaging?. J Nucl Med.

[36] Ignatovich ZV, Gusak KN, Chernikhova TV, Kozlov NG, Koroleva EV (2007). Interaction of secondary amines with aromatic aldehydes-efficient method for synthesis of the functionalized heterocyclic amines. Chem Heterocycl Compd.

[37] Node M, Hori H, Fujita E (1976) Demethylation of aliphatic methyl ethers with a thiol and boron trifluoride. J. Chem. Soc. Perkin Trans. I:2237–2240

[38] Košak U, Brus B, Gobec S (2014). Straightforward synthesis of orthogonally protected piperidin-3-ylmethanamine and piperidin-4-ylmethanamine derivatives. Tetrahedron Lett.

[39] Middleton WJ (1975). New fluorinating reagents. Dialkylaminosulfur fluorides. J Org Chem.

[40] Ellman GL, Courtney KD, Andres V, Featherstone RM (1961). A new and rapid colorimetric determination of acetylcholinesterase activity. Biochem Pharmacol.

[41] Mesulam M, Geula C (1994). Butyrylcholinesterase reactivity differentiates the amyloid plaques of aging from those of dementia. Ann Neurol.

[42] Meuling WJ, Jongen MJ, van Hemmen JJ (1992). An automated method for the determination of acetyl and pseudo cholinesterase in hemolyzed whole blood. Am J Ind Med.

[43] Jorissen WP, van der Beek PAA (1930). The oxidation of benzaldehyde. Recl Trav Chim Pays-Bas.

[44] Kabalka GW, Varma M, Varma RS, Srivastava PC, Knapp FF (1986). The tosylation of alcohols. J Org Chem.

[45] Mesulam MM, Guillozet A, Shaw P, Levey A, Duysen EG, Lockridge O (2002). Acetylcholinesterase knockouts establish central cholinergic pathways and can use butyrylcholinesterase to hydrolyze acetylcholine. Neuroscience.

[46] Reid GA, Chilukuri N, Darvesh S (2013). Butyrylcholinesterase and the cholinergic system. Neuroscience.

[47] Geula C, Nagykery N (2007). Butyrylcholinesterase activity in the rat forebrain and upper brainstem: postnatal development and adult distribution. Exp Neurol.

